# Acute Bilateral Vision Loss in a Young Male: A Case of Leber’s Hereditary Optic Neuropathy

**DOI:** 10.7759/cureus.86607

**Published:** 2025-06-23

**Authors:** Zineb Hilali, Oumaima El Korno, Younes Laarif, Noureddine Boutimzine, Lalla Ouafae Cherkaoui

**Affiliations:** 1 Faculty of Medicine, L'Hôpital des Spécialités de Rabat, Rabat, MAR; 2 Ophthalmology, Ibn Sina Hospital, Rabat, MAR; 3 Ophthalmology, L'Hôpital des Spécialités de Rabat, Rabat, MAR

**Keywords:** gene therapy, genetic disease, leber’s hereditary optic neuropathy, optic neuropathy, sudden vision loss

## Abstract

Leber’s Hereditary Optic Neuropathy (LHON) is a rare mitochondrial genetic disorder that primarily affects young adult males, leading to acute or subacute painless central vision loss. The condition results from point mutations in mitochondrial DNA, most commonly affecting the *ND1*, *ND4*, or *ND6* genes, which impair the function of complex I in the mitochondrial respiratory chain. This leads to selective degeneration of retinal ganglion cells and the optic nerve, causing severe and often irreversible visual impairment.

We present the case of a 24-year-old male farmer who consulted for rapidly progressive bilateral visual acuity loss. Visual acuity was measured at 1/10 in the right eye and “counting fingers at near” in the left eye, with a left relative afferent pupillary defect (RAPD). An extensive etiological workup for optic neuropathy was conducted. Genetic testing of the *MT-ND4* gene identified the 11778/ND4 mutation, confirming the diagnosis of LHON.

## Introduction

Leber’s hereditary optic neuropathy (LHON) is a rare genetic disorder caused by mutations in mitochondrial DNA. It primarily affects young adult men and leads to sudden or gradual painless loss of central vision. The disease is associated with mutations in the *ND1*, *ND4*, or *ND6* genes, which impair the function of complex I in the mitochondrial respiratory chain. This dysfunction leads to selective damage of the retinal ganglion cells and the optic nerve, resulting in severe and often permanent vision loss [1].

LHON typically presents with unilateral vision loss, which often progresses to the fellow eye within weeks to months. Early fundoscopic findings may include optic disc hyperemia, peripapillary telangiectasia, and retinal nerve fiber layer swelling, eventually progressing to optic atrophy [2].

## Case presentation

We report the case of a 24-year-old male farmer with no significant medical history who presented with painless, rapidly progressive bilateral vision loss, consistent with the typical presentation of LHON. Symptoms began in the left eye one and a half months prior, followed by involvement of the right eye one month ago, illustrating the sequential and asymmetric onset frequently observed in LHON.

On ophthalmologic examination, best-corrected visual acuity (BCVA) was 1/10 in the right eye (OD) and limited to counting fingers at near in the left eye (OS). The absence of ocular pain further supports a hereditary rather than inflammatory etiology. Anterior segment examination was unremarkable in both eyes. A relative afferent pupillary defect (RAPD) was noted in the left eye, indicating greater optic nerve dysfunction on that side.

**Figure 1:**
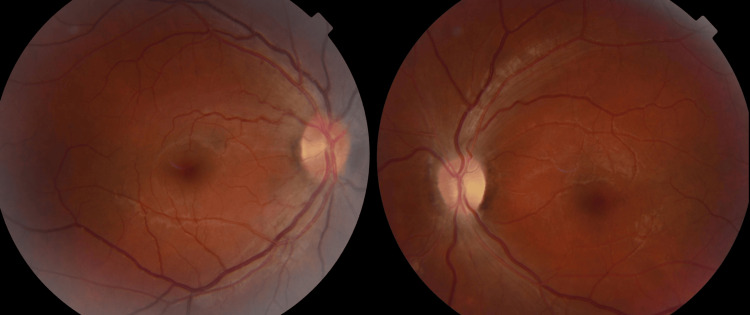
Fundus photography shows no evidence of papilledema.

Fundus examination showed papillary hyperemia in the right eye, suggestive of acute involvement, and optic disc pallor in the left eye, reflecting a more advanced, chronic stage. The foveal reflex was preserved, and retinal vessels were of normal caliber bilaterally (Figure 1). These findings support the classical clinical presentation of LHON.

**Figure 2:**
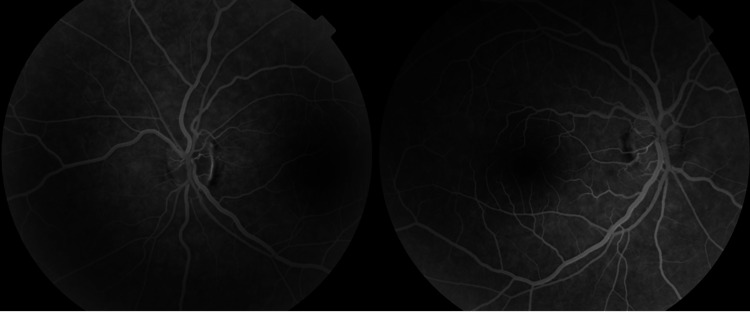
Fluorescein angiography shows no evidence of papilledema.

A retinal fluorescein angiography was performed and showed no late-phase optic disc leakage, ruling out inflammatory causes (Figure 2).

Optical coherence tomography (OCT) revealed peripapillary retinal nerve fiber layer (RNFL) thinning, consistent with optic nerve atrophy. The average RNFL thickness was 121 µm in the right eye (OD) and 70 µm in the left eye (OS), both below normal reference values (typically ~100-110 µm), with more advanced thinning in the left eye, correlating with the longer disease duration and worse visual acuity.

**Figure 3:**
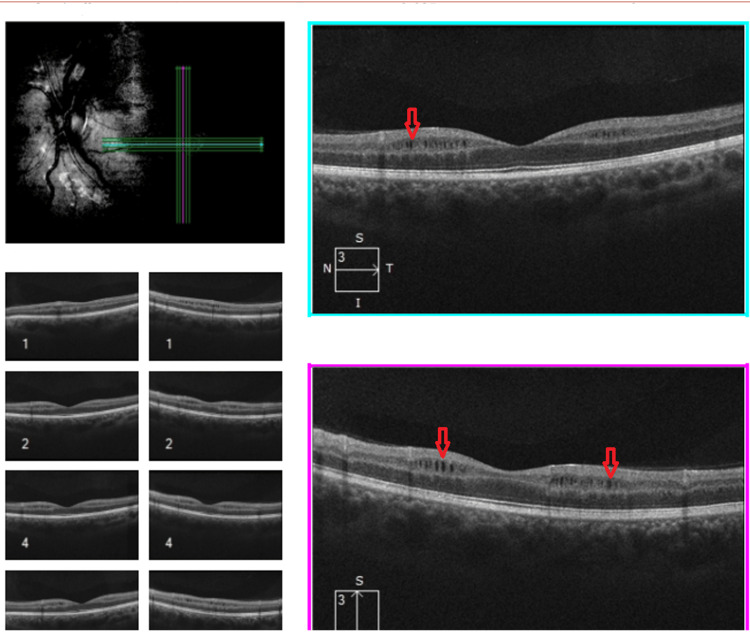
Macular OCT shows degenerative microcystic changes within the inner nuclear layer (red arrow), reflecting the extent of optic nerve damage. OCT: Optical coherence tomography.

Additionally, macular microcysts within the inner nuclear layer were noted bilaterally, a finding associated with chronic optic atrophy and potentially poorer visual prognosis in LHON. These structural changes (Figure 3), in the absence of optic disc leakage on fluorescein angiography, further support a diagnosis of non-inflammatory, hereditary optic neuropathy, helping to differentiate LHON from other inflammatory or demyelinating causes.

**Figure 4:**
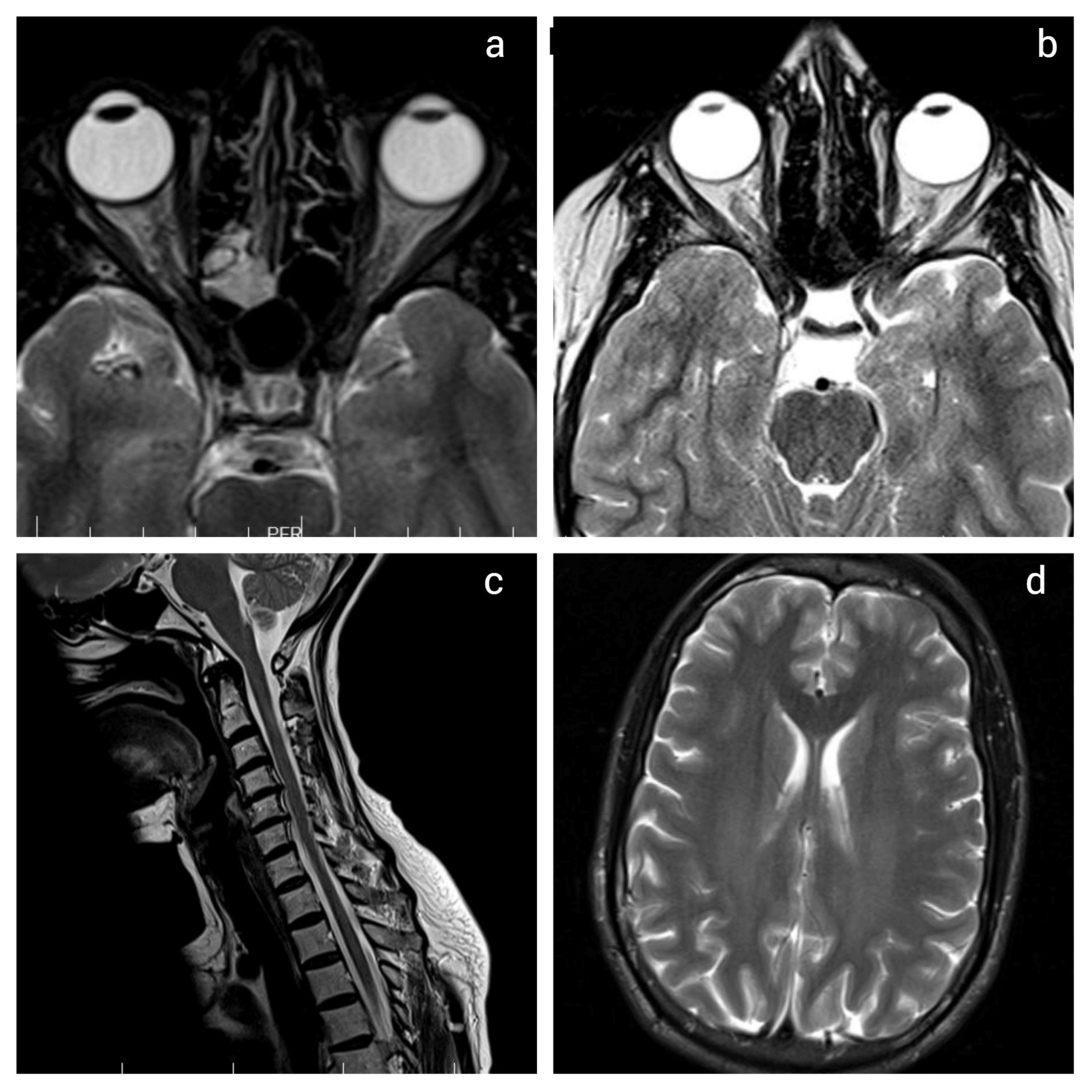
Orbital MRI focused on the optic nerves shows no signal enhancement or atrophy (a, b). Brain and spinal cord MRI in the T2 sequence reveal no abnormalities (c, d).

A comprehensive diagnostic workup for optic neuropathy was performed to rule out other potential causes. This included an orbital and brain MRI (Figure 4), complete blood count, serum electrolytes, calcium-phosphate panel, inflammatory markers, lumbar puncture with opening pressure, and a tuberculosis workup (chest X-ray, tuberculin skin test, GeneXpert, and QuantiFERON). Extensive infectious and autoimmune serologies were also conducted, including syphilis, HSV, VZV, CMV, EBV, HIV, HCV, HBV, and anti-AQP4 and anti-MOG antibodies. All investigations returned normal results, effectively excluding alternative diagnoses such as inflammatory, infectious, or demyelinating optic neuropathies.

Finally, genetic testing confirmed the presence of the m.11778G>A mutation in the *MT-ND4* gene, the most common pathogenic variant associated with LHON, thus establishing the definitive diagnosis.

## Discussion

LHON is a silent neurodegenerative disease that leads to acute or subacute irreversible central vision loss [1]. The condition predominantly affects young males (sex ratio 5:1), with peak onset between the ages of 15 and 30 [2]. The typical presenting symptom is acute or subacute unilateral visual loss, followed by sequential involvement of the fellow eye, usually within 6 to 8 weeks, and at most within one year [2]. Visual acuity typically reaches its lowest point within 4 to 6 weeks from the onset of symptoms, and in about 25% of cases, the vision loss may be bilateral from the beginning [2].

During the acute phase, clinical examination often reveals optic disc hyperemia associated with peripapillary telangiectatic microangiopathy, although fundus examination can appear normal in 20% to 40% of cases [1-3]. Fluorescein angiography typically shows no optic disc leakage, helping to distinguish LHON from inflammatory optic neuritis. Brain MRI is generally normal but may occasionally reveal hyperintensity of the optic nerve [4-5]. In the subacute to chronic phase, optic disc pallor develops, usually beginning temporally [5].

LHON was suspected in a young male patient with rapidly progressive, sequential bilateral vision loss. The clinical presentation was notable for a mismatch between OCT and fluorescein angiography findings, optic disc edema was observed on OCT, yet no corresponding leakage appeared on angiography. Bilateral macular microcysts were present, a finding reported in approximately 10% of LHON cases. The lack of response to corticosteroids, coupled with normal laboratory and imaging investigations, further supported a hereditary rather than inflammatory diagnosis [6-8].

LHON is a maternally inherited mitochondrial optic neuropathy caused by point mutations in mitochondrial DNA affecting complex I subunits of the respiratory chain. Around 90% of cases are linked to three primary mutations: m.11778G>A in ND4, m.3460G>A in ND1, and m.14484T>C in ND6. The disease shows incomplete penetrance, about 50% of male carriers and 10% of female carriers develop visual symptoms. Vision loss is driven by apoptotic degeneration of retinal ganglion cells, especially those in the papillomacular bundle (PMB) [7].

Environmental and lifestyle factors such as smoking, alcohol consumption, exposure to certain toxins, and the use of specific medications (e.g., antiretrovirals and antituberculosis drugs) may contribute to the onset or exacerbation of the disease in genetically predisposed individuals [1,6].

LHON typically progresses through four distinct stages. In the asymptomatic stage, subtle fundoscopic signs such as RNFL edema, peripapillary telangiectasias, and temporal thickening may be present, indicating early disease even in the absence of visual symptoms [1]. The acute stage is characterized by sudden, painless central vision loss, most often affecting one eye initially, with the fellow eye usually becoming involved within weeks to months. Ophthalmoscopic findings can include RNFL edema and damage to the PMB, although up to 30% of patients may exhibit no visible abnormalities during this phase. Visual acuity often deteriorates rapidly, accompanied by color vision defects (dyschromatopsia) and the development of central or centrocecal scotomas. During the subacute stage, retinal changes such as RNFL edema begin to resolve, although significant visual recovery remains rare. Finally, the chronic stage is marked by progressive optic atrophy and permanent vision loss. OCT typically reveals substantial RNFL thinning, particularly in the temporal quadrant, and most patients at this stage fulfill the criteria for legal blindness [7,8].

Several prognostic factors have been identified in LHON, providing insight into the likely severity and progression of visual loss. Among these, age at onset plays a significant role. Early-onset cases, particularly those diagnosed before the age of 12, are associated with a more favorable prognosis. The so-called infantile form of LHON appears to confer a degree of protection, with a higher likelihood of partial or even substantial visual recovery [8,9].

The type of mitochondrial mutation also greatly influences the visual outcome. Patients carrying the 14484/ND6 mutation have the best visual prognosis, with over half experiencing some degree of spontaneous recovery following the initial vision loss. In contrast, recovery is less common in individuals with the 11778/ND4 mutation (approximately 22%) and even rarer in those with the 3460/ND1 mutation (about 15.4%) [10]. 

One limitation of this case is the absence of family genetic testing. Although the identification of the m.11778G>A (MT-ND4) mutation in the index patient is sufficient to establish the diagnosis of LHON, family testing could have provided additional context regarding maternal inheritance patterns and penetrance within the family. Including such data would have strengthened the genetic and epidemiological understanding of the case.

Another notable factor is optic disc size. A larger optic disc has been correlated with better visual recovery and improved final visual acuity. Interestingly, this anatomical feature is more commonly observed in asymptomatic mutation carriers, suggesting that it may offer some resistance to the development of optic neuropathy in predisposed individuals [10].

The treatment of LHON starts with the strict elimination of environmental risk factors such as smoking, alcohol, toxins, and mitochondrial-toxic medications [10]. While various nutritional supplements have been tested, none have proven effective in large clinical trials [11].

The only treatment with demonstrated efficacy is idebenone, a synthetic CoQ10 analogue that supports mitochondrial function by bypassing complex I and reducing oxidative damage. It is most effective when started early, especially in young patients and those with 11778 or 3460 mutations. It is approved in Europe at 900 mg/day, taken for at least one year [11].

The LEROS study showed that idebenone remains effective even in chronic phases, if initiated within five years of symptom onset [11].

Another promising drug, EPI-743, is still under investigation but has shown potential benefits in visual recovery [11].

Additionally, gene therapy using Lenadogene nolparvovec (delivered via intravitreal injection) improved visual acuity in both eyes, likely due to trans-chiasmal viral transfer. The RESTORE study confirmed that vision can continue to improve for at least four years post-injection [1].

## Conclusions

Although historically considered untreatable, recent advances in molecular genetics and therapeutics have opened the door to potential treatments, including gene therapy and mitochondria-targeted interventions. Early diagnosis and genetic counseling are essential for effective patient management and informed family planning.
